# Detecting Encrypted and Unencrypted Network Data Using Entropy Analysis and Confidence Intervals

**DOI:** 10.3390/e25030397

**Published:** 2023-02-22

**Authors:** Oana-Adriana Ticleanu, Teodora Popa, Daniel Ioan Hunyadi, Nicolae Constantinescu

**Affiliations:** Department of Mathematics and Informatics, Faculty of Science, Lucian Blaga University of Sibiu, 550024 Sibiu, Romania

**Keywords:** encrypted data detection, data entropy, 11T71, 94A17, 28D20

## Abstract

The detection of clear and encrypted data that are transported through computer networks is of particular importance both for protecting the data and the users to whom they belong and to whom they are intended, as well as the networks through which they are transmitted. The proposed method consists of an algorithm that classifies the data it receives by testing the belongingness of their standard deviation values to established confidence intervals. Following the evaluation of the algorithm, an accuracy of 94.73% was obtained and it appears that the results can be used with certainty in subsequent analyses of the data detection.

## 1. Introduction

As data traffic on local and global networks has increased, so has data fraud and the need to analyze the types of data being communicated across networks. The detection of encrypted data and those in a clear format (data structures transmitted over a communication channel in unencrypted format, DSCT) that are transported through computer networks requires differentiating them correctly, using effective and up-to-date methods. The data identified in this way can be used for further analysis, facilitating the detection of unwanted situations and the determination of the safety level of the way in which they are transmitted and contributing to the sharing of data, their users, and the networks through which they are transported.

The identification of encrypted data that are transmitted over networks is useful because it allows their subsequent analysis and the detection of programs that compromise data and users, according to [1]. The detection of such programs also enables one to limit the negative effects on their actions.

Determining the data that are transmitted in the DSCT format through computer networks is valuable because it contributes to the identification of cases in which user data are transmitted in an unprotected manner. These data may contain personal details of the users, according to [2], where clearly transmitted data of some patients using devices from the Internet of Things (IoT) were detected.

To detect encrypted data and DSCT data, there are methods that consist of statistical approaches, methods that use automatic learning techniques, or methods that combine these two approaches.

The proposed method is intended to detect encrypted data and data in the DSCT format that are transported through the network, excluding data that are in unencrypted, but which are also compressed. This method consists of applying an algorithm that, for the data it receives and has to classify, estimates the entropies and calculates the standard deviation values. Based on the values of the standard deviations, for which the algorithm tests belonging to the established confidence intervals, the algorithm classifies the data as DSCT, encrypted, or unidentified data. In addition to the proposed algorithm, the way to determine the confidence intervals is also presented.

There are two confidence intervals:

- An interval for encrypted data;
- An interval for DSCT format.

In order to be able to determine these intervals, the data samples are divided into two categories: encrypted and DSCT data format. This classification was made based on partial entropy of each interval.

In this sense, the parameters that make up the characterization of a data flow are initially described. In the next step, the method of calculating the values that describe the entropy properties of the data is illustrated. These first two steps will be the basis of the calculation of the initial values that constitute the input data of the algorithm proposed in this article. After this, the algorithm and the comparative results obtained with it will be described, in addition to a description of its limitations as well as ways to customize it for other cases.

## 2. Methods for Detecting Encrypted and Unencrypted Data from the Network

As stated above, several methods of network traffic analysis have been developed in order to detect encrypted data as well as data transmitted in the clear. Each of the methods deals with a particular typology of data transferred within network transmissions.

Paper [3] notes both methods for identifying traffic that contains encrypted data, as well as approaches for detecting data in the DSCT format, that are transported: statistical approaches that analyze the distributions of transported data, methods that are based on the modes of interaction between hosts or the ports used by applications, and approaches that combine several methods.

The methods for detecting encrypted and unencrypted data presented in this section are statistical methods, those based on the use of automatic learning techniques, and methods that combine the two approaches.

### 2.1. Statistical Methods to Detect Encrypted and Unencrypted Data from the Network

#### 2.1.1. Using Entropy to Classify Data from the Network

Let $\Psi_{\phi_{E}}^{E}$ be a situation. This situation is described by some events that may or may not occur. For the description of situation $\Psi_{\phi_{E}}^{E}$, *N* events are provided. The set of events that can describe situation $\Psi_{\phi_{E}}^{E}$ is $E=\{\epsilon_{0},\epsilon_{1},\ldots ,\epsilon_{N-1}\}$. Each event in the set *E* has a probability of occurring. The set of probabilities of occurrence for each event from *E* is $\phi_{E}=\left\{\varphi_{\epsilon_{0}},\varphi_{\epsilon_{1}},\cdots ,\varphi_{\epsilon_{N-1}}\right\}$.

In his work, Shannon [4] proposes Equation (1), which he calls entropy and by which he represents the uncertainty of the procedure of the events in the set *E*, which have the probabilities of occurrence in the set $\psi_{E}$ and which describe the situation $\Psi_{\phi_{E}}^{E}$:

(1) $$\Omega \left(\Psi_{\phi_{E}}^{E}\right)=-\sum_{t=0}^{N-1}\varphi_{\epsilon_{t}}\cdot log\left(\varphi_{\epsilon_{t}}\right),\epsilon_{t}\in E,\varphi_{\epsilon_{t}}\in \phi_{E}$$

According to [4], the most uncertain situation is when all the elements of the set $\psi_{E}$ have the same value. Entropy helps to detect encrypted and unencrypted data in the network. In this case, as in [1], the entropy measurement unit is the bit, and the logarithm has a base equal to 2. Thus, the entropy will have the formula:

(2) $$\Omega \left(\Psi_{\phi_{E}}^{E}\right)=-\sum_{t=0}^{N-1}\varphi_{\epsilon_{t}}\cdot log_{2}\left(\varphi_{\epsilon_{t}}\right),\epsilon_{t}\in E,\varphi_{\epsilon_{t}}\in \phi_{E}$$

The calculation of Shannon’s entropy value assumes, as noted in [4], that the elements of the set $\Psi_{\phi_{e}}^{E}$ are known: for each event $\varphi_{\epsilon_{t}}$ describing the situation $\epsilon_{t}$, the probability of $\varphi_{\epsilon_{t}}$ occurring is known: $t=\bar{0,N-1}$.

As it is described in other previous research, to compute the entropy value in this way is impossible in practical cases from data flows transferred along communication networks because it requires a lot of data that are impossible to obtain in the case of a real situation, such as the detection of encrypted data and clear data in such communications.

Thus, alternative methods of using entropy have appeared for detecting encrypted data and those in the DSCT format, two of which are entropy value estimation and entropy truncation.

*Estimation of the entropy value*

According to work [1], it is possible to exactly define the parameters that compose the description of situations of the type $\Psi_{\phi_{e}}^{E}$.

Let $\eta_{E}$ be the number of events that are effectively involved in the description of the situation $\Psi_{\phi_{E}}^{E}$ and $\eta_{\epsilon }$ the set of numbers of occurrences of the events that describe the situation $\Psi_{\phi_{E}}^{E}$. The set $\eta_{\epsilon }$ is $\eta_{\epsilon }=\left\{\eta_{\epsilon_{0}},\eta_{\epsilon_{1}},\ldots ,\eta_{\epsilon_{N-1}}\right\}$ and has the property of (3): the sum of all elements of the set $\eta_{\epsilon }$ is equal to $\eta_{E}$.

(3) $$\sum_{t=0}^{N-1}\eta_{\epsilon_{t}}=\eta_{E},\;\eta_{\epsilon_{t}}\in \eta_{\epsilon },\;\epsilon_{t}\in E$$

Let the event that describes the situation $\Psi_{\phi_{E}}^{E}$ have a frequency defined according to (4):

(4) $$\vartheta_{\epsilon_{t}}=\frac{\eta_{\epsilon_{t}}}{\eta_{E}},\;t=\bar{0,N-1},\;\epsilon_{t}\in E,\;\eta_{\epsilon_{t}}\in \eta_{\epsilon }$$

There is a range of different methods by which the entropy value can be estimated. In accordance with the same work, among these values are the maximum likelihood estimation of the entropy value, the Miller–Madow entropy estimation, and the sheared version of the maximum likelihood estimation.

According to [5], the maximum probability estimation applied to the entropy of situation $\Psi_{\phi_{e}}^{E}$ has the formula

(5) $$\Omega_{max\psi }\left(\Psi_{\phi_{E}}^{E}\right)=-\sum_{t=0}^{N-1}\vartheta_{\epsilon_{t}}\cdot log_{2}\left(\vartheta_{\epsilon_{t}}\right),\;\epsilon_{t}\in E,\;\vartheta_{\epsilon_{t}}=\frac{\eta_{\epsilon_{t}}}{\eta_{E}},\;\eta_{\epsilon_{t}}\in \eta_{\epsilon }$$

Let $\eta_{\psi_{E}}\neq 0$ be the number of events in *E* that describe situation $\Psi_{\phi_{E}}^{E}$ and that have a probability of occurrence different from 0. As noted in [5], the Miller–Madow estimation takes into account and number $\eta_{\psi_{E}}\neq 0$ having the formula

(6) $$\Omega_{Miller-Madow}\left(\Psi_{\phi_{E}}^{E}\right)=\Omega_{max\psi }\left(\Psi_{\phi_{E}}^{E}\right)+\Delta ,\;\Delta =\frac{\eta_{\psi_{E}}\neq 0}{2\eta_{E}ln\left(2\right)}$$

The sheared version of the maximum likelihood estimation applied to the entropy has the form in Equation (7), as was proposed in [1]:

(7) $$\Omega_{Fmax\psi }\left(\Psi_{\phi_{E}}^{E}\right)=\eta_{E}\cdot \Omega_{max\psi }\left(\Psi_{\phi_{E}}^{E}\right)-\frac{\eta_{E}-1}{\eta_{E}}\cdot \sum_{\upsilon =0}^{\eta_{E}-1}\Omega_{max\phi }\left(\Psi_{\phi_{E}\backslash \left\{\varphi_{\epsilon_{j}}\right\}}^{E\backslash \{\epsilon_{\upsilon }\}}\right)$$

*Entropy truncation*

The entropy truncation algorithm for the available sample consists of a series of complex operations, and the necessary formula to compute the truncated entropy value is described in [5] as

(8) $$\Omega \left(\Psi_{\phi_{e}}^{E}\right)=\frac{1}{{\left(N\right)}^{\eta_{E}}}\cdot \sum_{\eta_{\epsilon }}\left(\eta_{E}\cdot \prod_{t=0}^{N-1}\frac{1}{(\eta_{\epsilon_{t}})!}\right)\cdot \left(\Omega_{max\phi }\Psi_{\phi_{E}}^{E}\right)$$

In this way, entropic analysis is used to analyze data from the network and to classify them into encrypted and clear data as well as an encrypted traffic detector based on the first packet in the flow and the data contained in it. In [6], it was proved that entropy can accurately determine whether traffic data are encrypted or not, this being used for outgoing traffic.

*Applying entropy to detect encrypted data and clear data from the network*

According to previous formulas, their proposed method to detect encrypted data and clear data using entropy has two steps:

- Calculation of a threshold value using entropy truncation for the available sample (Equation (8));
- Estimation of the entropy value with Equation (9) (maximum probability estimation) for the data to be detected; comparing the estimated values and the threshold values.

There are several methods to compute the threshold value; some of them use the Monte Carlo method, others use the confidence intervals to differentiate the encrypted data from the clear data.

#### 2.1.2. The Use of Other Statistical Methods to Detect Encrypted Data and Clear Data from the Network

In work [2], it was proposed to apply the $\chi^{2}$ test, which consists of the comparison of frequencies and uses Equation (9) for the detection of encrypted and clear data.

(9) $$\chi^{2}\left(\Psi_{\phi_{E}}^{E}\right)=\sum_{t=0}^{N-1}\frac{{\left(\vartheta_{\epsilon_{t}}-\vartheta_{\omega u_{t}}\right)}^{2}}{\vartheta_{\omega u_{t}}},\;\vartheta_{\omega u_{t}}=\mathrm{the}\;\mathrm{frequency}\;\mathrm{of}\;\mathrm{the}\;\mathrm{uniform}\;\mathrm{distribution}$$

In [7], the author proposed using the test for autocorrelation and the Kolmogorov–Smirnov test in order to ameliorate the detection accuracy. In [8], the authors proposed a combination of a set of tests: Anderson–Darling and Poker tests and also the tests from the NIST series are mentioned as a solution to improve the accuracy of the detection of encrypted data and data in the clear format. Among the limitations of these models are those related to the fact that the analysis can only be done for certain types of data that are transferred within the communication systems. This fact also emerges from the studies carried out within these studies, which highlight the structure of the data analyzed in the practical testing of the proposed models. In this sense, various studies are continuously undertaken to be applicable for the cases required in certain types of communications. At the same time, the studies that refer to a general framework of data transmissions are in progress.

### 2.2. Methods That Use Machine Learning to Detect Encrypted Data and Those in the Clear Form

In [9], following the application of entropy estimation, a support vector machine model was used to remove unencrypted traffic and keep encrypted traffic. In this way, applying from the network packets, the use of a machine learning type classifier is taken into account, but this only to do a certain filtering of the data transmitted in encrypted mode. Compressed data are overlooked, using a binary entropy relief method. Through this method, particular cases of executables that are likely to contain malware viruses are studied. The method actually highlights the traffic that has a high entropy, thereby classifying the data of interest for this study. In order to improve the training time of the network, the authors use an algorithm for the selection of certain features of interest, features that are detected from several algorithms that determine the entropy. The best results for the studies done in this sense were obtained by combining a support vector machine (SVM) algorithm with a personal method, developed by them, through which certain features of interest are extracted.

In continuation of these studies, other approaches outline that the automatic learning is useful for the analysis of already classified traffic, as in [10], in which the following automatic learning methods are addressed for the analysis of encrypted traffic chosen after entropy analysis: support vector machine, random forest, naive Bayes classifier, logistic regression, and neural networks. The maximum percentage of detectability, in ideal cases, was 89.70%.

In addition to these, automatic learning can help in the case of detecting clear and compressed data and differentiating them from encrypted ones; however, it again has to be specified that these are applicable only for certain cases.

### 2.3. In-Depth Statistical Parameters, Used in Neural Networks

In [11], an architecture based on neural networks is used to differentiate between encrypted and compressed (but clear) data. Starting from the combination of some statistical parameters, certain values are selected to be used in a larger test package, which will determine the input parameters in a particular machine learning model, based on rectified linear unit activation (ReLU) and scaled exponential linear unit activation (SeLU) functions. The assumed results refer to certain well-defined lengths of the data to be analyzed, representing a limitation of the proposed system. A way to determine the type of data from the content is presented, namely the one based on the $\chi^{2}$ test, for the sample value of 256 (from 0 to 255), thereby highlighting the fact that the storage of the analyzed data is made for coding lengths of 8 bits: $\chi^{2}=\sum_{i=0}^{255}\frac{{\left(N_{i}-E_{i}\right)}^{2}}{E_{i}}$.

**Figure 1:**
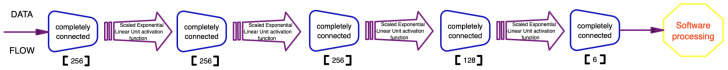
Neural network logical scheme.

What is interesting in this study is how the neural network architecture is defined. From Figure 1, it can be seen that the way in which the $\chi^{2}$ function was used has an influence on the architecture of the neural network, which highlights the fact that it can only be used on a system that uses certain particular data types of transmissions, which significantly reduces the area of use of the results in practical applications. The maximum percentage of detectability, in ideal cases, was 92%.

### 2.4. The Nearest Neighbor Method

In [12], the nearest neighbor method, forward propagation neural networks, and convolutional neural network are proposed for separating encrypted data from clear but compressed data; the best results coming with the convolutional neural network.

The presented model starts from the calculation of the neighborhood characteristics between various parts of the data and from them extracts a model that can be used in a neural network. A limitation at this point of the research is given by the fact that it is necessary to manually identify the characteristics that will be parameterized as system inputs. As with the previously presented studies, the $\chi^{2}$ characteristic is used, which will compare each value obtained with a certain reference characteristic, encoding each analyzed packet in an associated data vector. The authors admit that it is necessary to study more efficient methods to determine such characteristics, but if such ways are approached, the method would also consume a lot of computing resources and long learning processes, which must be manually assisted. The maximum percentage of detectability, in ideal cases, for the best version of the system, was 66.90%.

The limitations of this system refer to the fact that the proposed solution will be able to determine the compressed text, but transmitted in a clear, unencrypted format, for small traffic, within Internet of Things networks.

### 2.5. Using the Vector Machine Model on Local Entropy

Paper [13] proposed a method that uses an algorithm to extract a particular sequence of bits of a well-defined length. In successive steps, this sequence is offset so that a certain area of the analyzed data can be covered. From these sequences, the associated entropies are calculated and a vector of them is determined, which will be used to parameterize the vector machine support system. The proposed model is evaluated only for the particular case where the packets have a length of 1444 bytes.

The maximum percentage of detectability, only for particular cases when the packets have a length of 1444 bytes, was 97.90%, but for most cases, the average percentage of detectability was approximately 59%, and the maximum obtained for certain types of particular data was 72%.

### 2.6. Parameter Estimation Using the Monte Carlo Method

In [14], a three-step solution was proposed. In the first step, the packages that are analyzed are classified, and some relations between the types of packages and the reception times are created. In the second step, the packets that exceed the length of 1024 bytes are determined, and these are the objects of the analysis. In the third step, a simulation applying the Monte Carlo method is used to compute the coordinates of a number of circles that will constitute the elements to be analyzed, in the sense that for each of their intersections, an estimate of the type π for the intersecting circles is determined. The method actually constitutes a solution for computing the approximations between the entropies of the sequences analyzed from the simulation: a neighborhood analysis, similar to the previous methods, but using a larger number of data by generating them using the Monte Carlo method. By this it can be deduced that this solution can be used to determine some approximations if data traffic is analyzed for a short period of time, therefore reducing the data flow.

The percentage of detectability, considering the average for the three chosen types, was 92.49%, and the maximum was 94.98%, this value being obtained for a particular case.

*Solutions overview*

Because there is ever-increasing activity of attack actions on the networks of electronic devices, the analysis of network traffic is a desideratum that is transposed by the importance given to research in the field. There are a multitude of results that each deal with a different way of doing this detection, each of the proposed solutions approaching a technique to improve the already existing methods for various types of data traffic. The research community is still discovering generally valid methods that can be used for any type of data flows, from any communication system. The method approached in the current research is based on the idea of doing a successive analysis of the data, analyzing their entropy, and transferring the resulting parameters into a form that can be analyzed statistically. What can be highlighted from the analyzed cases is that all the proposed solutions have low detectability rates for general cases and efficiency in very particular cases. By this, in the method proposed in the present article, current techniques are combined and methods are added to select certain properties that define the encrypted data.

## 3. The Proposed Method for Detecting Encrypted Data and Clear Data from the Network

The proposed method for differentiating the encrypted data from the plain data consists of:

- Generating some confidence intervals that can be used to detect encrypted data and plain data;
- Proposing an algorithm that estimates the entropy value and the way to compute the standard deviation for the data that has to be classified, placing the data in the appropriate category based on the standard deviation belonging to the confidence intervals;
- Evaluating the proposed algorithm.

**Figure 2:**
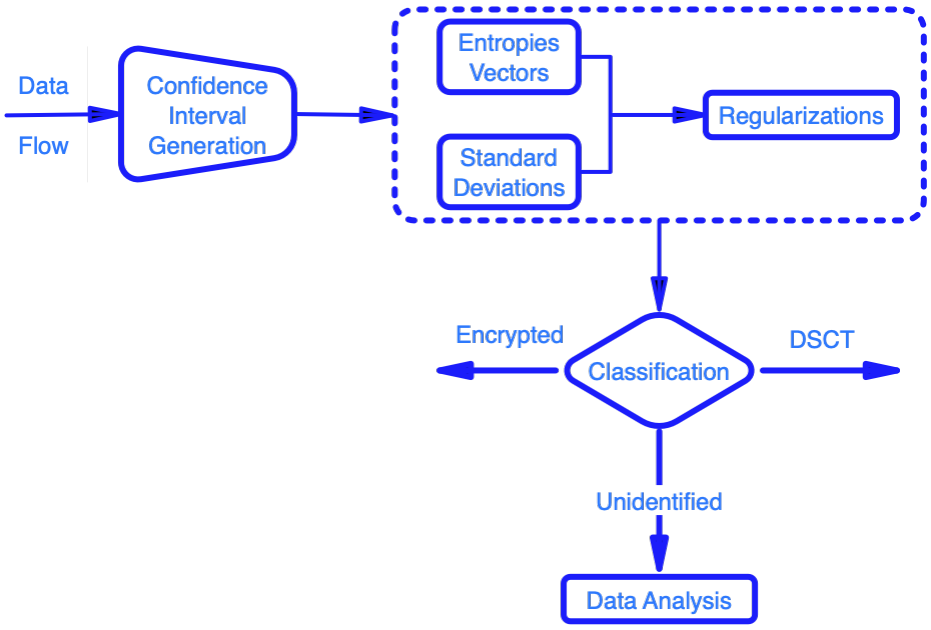
Proposed solution scheme.

The manner in which the proposed solution is approached as well as its component parts are schematically described in Figure 2.

Beyond this, an analysis of the limitations induced by the model is completed.

### 3.1. Generation of Confidence Intervals Used in the Detection of Encrypted Data and Data in the Clear From

To determine the two confidence intervals, ${ℑ}_{Cr}^{\sigma }$ for the detection of encrypted data and ${ℑ}_{Cl}^{\sigma }$ for the detection of data in the DSCT format, we used two samples:

- A sample containing only encrypted data used to determine the ${ℑ}_{Cr}^{\sigma }$ range;
- The other sample containing only clear data used to determine the ${ℑ}_{Cl}^{\sigma }$ interval.

The two samples were generated using a traffic generator.

To determine the confidence intervals, the truncated entropy value is calculated according to Equation (8) for each of the two samples. The two resulting values are used to determine the extremes of the two intervals.

Thus, the two intervals were generated as defined in Equation (10) for the interval that determines the existence of encrypted data and Equation (11) for the interval that marks the existence of clear data in the network.

(10) $${ℑ}_{Cr}^{\sigma }=\left[\alpha_{Cr},\beta_{Cr}\right]=\left\{x\in R,\;\alpha_{Cr}\leq x\leq \beta_{Cr}\right\}$$

(11) $${ℑ}_{Cl}^{\sigma }=\left[\alpha_{Cl},\beta_{Cl}\right]=\left\{x\in R,\;\alpha_{Cl}\leq x\leq \beta_{Cl}\right\}$$

Both intervals are closed and bordered on both sides.

### 3.2. Detection of Encrypted Data and Clear Data from the Network with the Help of Confidence Intervals

As in [1,10], the proposed method estimates the entropy value by estimating the maximum probability for the data to be classified.

This value is then used to compute the standard deviation value. The value of the standard deviation determines whether the analyzed data are encrypted or in the clear.

Let σ be the standard deviation for the analyzed data. The analyzed data are encrypted (confidence level 96%) if condition (12) is satisfied and condition (13) is not satisfied. The analyzed data are clear (confidence level 96%) if condition (13) is satisfied and condition (12) is not satisfied at the same time.

(12) $$\sigma \in {ℑ}_{Cr}^{\sigma }\Rightarrow \sigma \in \left[\alpha_{Cr},\beta_{Cr}\right]\Rightarrow \alpha_{Cr}\leq \sigma \leq \beta_{Cr}$$

(13) $$\sigma \in {ℑ}_{Cl}^{\sigma }\Rightarrow \sigma \in \left[\alpha_{Cl},\beta_{Cl}\right]\Rightarrow \alpha_{Cl}\leq \sigma \leq \beta_{Cl}$$

If the standard deviation σ does not satisfy either condition (12) or (13) or satisfies both of them simultaneously, then the data are considered unidentified.

### 3.3. The Proposed Algorithm for Detecting Encrypted Data and Clear Data from the Network

Starting from the methods to compute the parameters, according to those described above, an algorithm named VTA (Algorithm 1) was established that proposes a combined method of classification of encrypted data, of those transferred in the clear, of DSCT type, and of unidentified ones.
**Algorithm 1** Message Classification: Encrypted—DSCT—Unidentified—VTA1:Initialize $M_{Cr},M_{Cl}$ and $M_{N}$ as empty sets2:**for** each $\mu_{t}\in M$ **do**3:      encrypted = FALSE4:      clear = FALSE5:      $\Omega_{max\varphi }\left(\mu_{t}\right)$ = Estimate_Entropy $\left(\mu_{t}\right)$6:      $\sigma_{\mu_{t}}$ = Compute_Standard_Deviation $\left(\mu_{t},\;\Omega_{max\varphi }\left(\mu_{t}\right)\right)$7:      **if** $\sigma_{\mu_{t}}\in {ℑ}_{Cr}^{\sigma }$ **then**8:          encrypted = TRUE9:      **end if**10:    **if** $\sigma_{\mu_{t}}\in {ℑ}_{Cl}^{\sigma }$ **then**11:        clear = TRUE12:    **end if**13:    **if** encrypted==TRUE **then**14:        **if** clear==TRUE **then**15:           $M_{N}$.add($\mu_{t}$)16:        **else** $M_{Cr}$.add($\mu_{t}$)17:        **end if**18:    **else**19:        **if** clear==TRUE **then**20:           $M_{Cl}$.add($\mu_{t}$)21:        **else** $M_{N}$.add($\mu_{t}$)22:        **end if**23:    **end if**24:**end for**

The proposed algorithm has as input data a lot of messages, about which it is not known whether they are encrypted or in the clear, but it is known that each message is either encrypted or in the clear.

Let *M* be the set of messages: $M=\{\mu_{0},\ldots ,\mu_{p-1}\}$, where *p* is the number of messages, and $\mu_{t}\in \left\{\mathrm{encrypted},\mathrm{clear}\right\},\;t=\bar{0,p-1}$.

Along with the multitude of messages, the algorithm also receives the two confidence intervals ${ℑ}_{Cr}^{\sigma }$, ${ℑ}_{Cl}^{\sigma }$.

Let $M_{Cr}$ be a set that is initially empty and that is populated by the algorithm with encrypted messages during data analysis and $M_{Cl}$ be a set that is initially empty and that is populated during the execution of the algorithm with messages identified as clear messages. Let $M_{N}$ be a set that is initially empty and that is populated when the algorithm runs with the messages considered unidentified. The properties of the three sets are relations (14) and (15).

(14) $$M=M_{Cr}\cup M_{Cl}\cup M_{N}$$

(15) $$⌀=M_{Cr}\cap M_{Cl}\cap M_{N}$$

Properties (14) and (15) are satisfied because the three sets are populated only with messages from *M* and not others. All messages in *M* must be assigned to a single set.

For each message $\mu_{t},\;t=\bar{0,p-1}$ in *M*, the following operations take place: applying the maximum probability estimate to the entropy, using Equation (5) and resulting value $\Omega_{max\varphi }\left(\mu_{t}\right)$; calculating the value of the standard deviation using the $\Omega_{max\varphi }\left(\mu_{t}\right)$ value, resulting in $\sigma_{\mu_{t}}$; fitting the $\sigma_{\mu_{t}}$ value into the corresponding intervals/the corresponding interval; inserting the message $\mu_{i}$ into the related set $M_{Cr},\;M_{Cl}$, or $M_{N}$. The operations are performed on all bits of the $\mu_{t}$ message.

The Estimate_Entropy function returns the estimated value of the entropy using the maximum probability estimate, computed according to Equation (5), for the message $\mu_{t},t=\bar{0,p-1}$.

The Compute_Standard_Deviation function computes the value of the standard deviation of the messages $\mu_{t},\;t=\bar{0,p-1}$ based on the estimated value of the entropy $\Omega_{max\varphi }\left(\mu_{t}\right),\;t=\bar{0,p-1}$.

### 3.4. Evaluation of the Proposed Algorithm for Detecting Encrypted Data and Clear Data in the Network

To evaluate the proposed algorithm, three types of tests were performed to see the functionality in the ideal case, in cases of conglomerate traffic (from various known sources), and in the case of general traffic. In this sense, the types of testing used consisted of:

**Case One:**  Generation of a number of 10,000 messages, of which 5000 were made up of encrypted data and 5000 of DSCT type data;
**Case Two:**  Analysis of 5000 items from data flow, where the applications that generate the data traffic are known;
**Case Three:**  Analysis of 5000 items from data flow from general data traffic, without having knowledge about the applications that generate the traffic. This type of analysis can be adapted to have applicability in other fields as well, such as for the studies done in [15,16,17]. In this sense, collaborations were opened with researchers in the field.

To classify a message as encrypted or clear (DSCT type), there are all four fault possibilities for each case: false positive, false negative, true positive, and true negative. This method of evaluation represents one of the key differences between the approach in the proposed solution and similar approaches, because the latter only deal with the case of non-identification of a data set type in a given context.

To mark a message as identified, neither true positive nor false negative results are expected, because no message from the input data of the algorithm is generated to be unidentified: any message in *M* is encrypted, or is in the DSCT format.

### 3.5. Evaluation for Case One

A total volume of ten thousand datasets were analyzed in the case where the data were generated by a traffic generator and were of the encrypted or DSCT type. The following abbreviations were used:

**TVAD**  The total volume of analyzed data;
**CV**  Classification volume;
**PCT**  Percentage of correct detections for VTA proposed algorithm;
**DSCT**  Data structures transmitted over a communication channel in unencrypted format;
**True Positive e.**  True positive—encrypted (correct detection);
**True Positive c.**  True positive—in clear (correct detection);
**Encrypted False p.**  Encrypted false positives (actually: DSCT);
**False Positive c.**  False positives—clear (actually: encrypted);
**False Positive u.**  False positives—unidentified (actually: DSCT).

The comparative evaluation was done along with five other typical solutions. The limitations of the comparison are given by the fact that some solutions only describe the correct detections and do not treat other cases: they do not take into account uncertainly determined data and the types of data chosen for analysis are particular. The alternative solutions considered for the comparative evaluation, as well as the particularities of the test, are described below:

**DbScan:**  A clustering algorithm. For this case, the maximum detection percentage of the solution was taken into account for the ideal case of analyzed data. The data were taken from the tests presented in [9].
**LibSVM:**  A library of functions used in the detection of encrypted data. It is based on the support vector machine algorithms. The detection case for ideal situations was considered. The data were taken from the tests presented in [9].
**EnCod:**  A solution proposed in [11]. It considered the ideal data case, treated in the cited article.
**k-NN:**  A solution proposed in [12]. Data for the ideal case were considered.
**SBE:**  A solution proposed in [13]. The data for the ideal case were considered, namely that of known data, more precise audio files, and the length of the data as specified in the study as optimal.

The results are described in Table 1.

### 3.6. Evaluation for Case Two

In this case, for the proposed PCT algorithm, a set of ten thousand data generated by known applications were used, and for the algorithms considered in the comparison, the data given for cases of medium complexity were taken. The limitations of the comparison reside in the fact that some solutions only describe the values for particular cases, suitable for those types of solutions. The particularities of the solutions considered in the comparative test are:

**DbScan:**  The data were taken from the tests presented in [9].
**LibSVM:**  Results for data of known types were considered. The data were taken from the tests presented in [9].
**EnCod:**  This considered the data of known types, treated in [11].
**k-NN:**  This considered the data of known types, treated in [12]. The authors used 3000 training packets and 700 test packets.
**SBE:**  This considered the data of known types, treated in [13].

The results are described in Table 2.

### 3.7. Evaluation for Case Three

For this studied case, for the proposed PCT solution, general traffic was analyzed, without having a priori knowledge about it. Not all the solutions considered for the comparative test have studied this case, which is the most difficult to analyze by such models. The types of data considered for all the comparative solutions were the general ones, without a priori knowledge. For the proposed EnCod solution, the authors do not treat the general case. For k-NN, the authors used 3000 training packets and 700 test packets. For SBE, the authors considered data of known type.

The results are described in Table 3.

## 4. Limitations of the Proposed Model

The proposed solution works, like other existing solutions, with a good accuracy rate. However, for Case Three, treated in Section 3.7, the required computing power is considerable. Therefore, for an analysis of the traffic from a network node, the implementation of the solution is suitable only for a high-power server, which limits its use only for some companies. For the case of private use, the price of such systems becomes prohibitive.

## 5. Conclusions and Future Work

The present paper proposed a method based on the entropic analysis of data for the detection of encrypted and clear messages in the network. The chosen method is statistically efficient and generates correct confidence intervals based on known samples, computation of the standard deviation, framing the value of the standard deviation in the confidence intervals, and classification of messages. The proposed model detects with an accuracy that varies between 97.97% and 63.40%, for the general case, the encrypted data, and the DSCT format from the network.

According to what is described in Section 4, high computing power is required in order to deal with the case of data without a known format, therefore the main goal of future studies is to improve the accuracy of the algorithm for Case Three (described in Section 3.7). For this, collaborations have been started for another variants, to implement evaluation systems of some additional entropy estimation parameters, according to the studies from [18,19]. Beyond this, as previously stated, is in our attention to adapt the current version to a solution that is suitable for implementation on low-power computing machines.

## Figures and Tables

**Table 1:** Case One—known data type, only encrypted and DSCT.

Message Type	TVAD	Classification	CV	PCT	DbScan	LibSVM	EnCod	k-NN	SBE
Encrypted	5000	True Positive e.	4895	97.97%	89.70%	96.63%	94%	66.9%	97.90%
		True Positive c.							
		Encrypted False p.	26						
		False Positive c.	52						
		False Positive u.	27						
DSCT	5000	True Positive e.	4902						
		True Positive c.							
		Encrypted False p.	24						
		False Positive c.	22						
		False Positive u.	52						

**Table 2:** Case Two—known data type.

Message Type	TVAD	Classification	CV	PCT	DbScan	LibSVM	EnCod	k-NN	SBE
Encrypted	5000	True Positive e.	4588	93.05%	89.7%	86.95%	92%	60%	72%
		True Positive c.							
		Encrypted False p.	254						
		False Positive c.	124						
		False Positive u.	34						
DSCT	5000	True Positive e.	4717						
		True Positive c.							
		Encrypted False p.	119						
		False Positive c.	72						
		False Positive u.	92						

**Table 3:** Case Three—general data type.

Message Type	TVAD	Classification	CV	PCT	DbScan	LibSVM	EnCod	k-NN	SBE
Encrypted	5000	True Positive e.	3051	63.40%	63%	61%	-	58.5%	65%
		True Positive c.							
		Encrypted False p.	328						
		False Positive c.	324						
		False Positive u.	1297						
DSCT	5000	True Positive e.	3289						
		True Positive c.							
		Encrypted False p.	284						
		False Positive c.	217						
		False Positive u.	1210						

## Data Availability

Not applicable.

## Abbreviations

The following abbreviations are used in this manuscript:

IoT	Internet of Things
DSCT	Data structures (messages) transmitted over a communication channel in unencrypted format
ReLU	Rectified linear unit activation function
SeLU	Scaled exponential linear unit activation function

## References

[1] Goubault-Larrecq J., Oivain J. (2006). Detecting Subverted Cryptographic Protocols by Entropy Checking.

[2] Wood D., Apthorpe N., Feamster N. Cleartext Data Transmissions in Consumer IoT Medical Devices. Proceedings of the 2017 Workshop on Internet of Things Security and Privacy (IoTS&P ’17).

[3] Cha S., Kim H. Detecting Encrypted Traffic: A Machine Learning Approach. Proceedings of the 17th International Workshop (WISA 2016).

[4] Shannon C.E. (1948). A Mathematical Theory of Communication. Bell Syst. Tech. J..

[5] Dorfinger P. (2010). Real-Time Detection of Encrypted Traffic Based on Entropy Estimation. Master’s Thesis.

[6] Exfild F.T.W. (2010). A Tool for the Detection of Data Exfiltration Using Entropy and Encryption Characteristics of Network Traffic. Master’s Thesis.

[7] Malhotra P. (2007). Detection of Encrypted Streams for Egress Monitoring. Master’s Thesis.

[8] Casino F., Choo K.-K.R., Patsakis C. (2019). HEDGE: Efficient Traffic Classification of Encrypted and Compressed Packets. IEEE Trans. Inf. Forensics Secur..

[9] Mamun M.S.I., Ghorbani A.A., Stakhanova N. An An Entropy Based Encrypted Traffic Classifier. Proceedings of the 17th International Conference on Information and Communications Security (ICISC 2015).

[10] Zhou K., Wang W., Wu C., Hu T. (2020). Practical evaluation of encrypted traffic classification based on a combined method of entropy estimation and neural networks. Etri J. Wiley.

[11] De Gaspari F., Hitaj D., Pagnotta G., De Carli L., Mancini L.V. (2022). Reliable detection of compressed and encrypted data. Neural Comput. Appl..

[12] Hahn D., Apthorpe N., Feamster N. Detecting Compressed Cleartext Traffic from Consumer Internet of Things Devices. arXiv.

[13] Tang Z., Zeng X., Sheng Y. (2019). Entropy-based feature extraction algorithm for encrypted and non-encrypted compressed traffic classification. Int. J. Innov. Comput. Inf. Control.

[14] Zhai J., Shi H., Wang M., Sun Z., Xing J. (2020). An Encrypted Traffic Identification Scheme Based on the Multilevel Structure and Variational Automatic Encoder. Secur. Commun. Netw..

[15] Lavinia D., Elisabeta A., Maria T., Mihail P., Calina S.S. (2020). Contribution of mechanical and electrical cardiovascular factors in patients with ischemic stroke. Pak. J. Pharm. Sci..

[16] Calina S.S., Elisabeta A., Lavinia D. (2020). The importance of balance and postural control in the recovery of stroke patients. Balneo Res. J..

[17] Dutescu M.M., Popescu R.E., Balcu L., Duica L.C., Strunoiu L.M., Alexandru D.O., Pirlog M.C. (2019). Social Functioning in Schizophrenia Clinical Correlations. Curr. Health Sci. J..

[18] Acu A.M., Maduta A., Otrocol D., Rasa I. (2020). Inequalities for Information Potentials and Entropies. Mathematics.

[19] Acu A.M., Hodis S., Rasa I. (2020). Estimates for the Differences of Certain Positive Linear Operators. Mathematics.

